# Effect of COVID-19 response in Uganda on street children

**DOI:** 10.11604/pamj.supp.2020.35.2.23545

**Published:** 2020-05-29

**Authors:** Brenda Allen Kawala, Brian Kibiwott Kirui, Samuel Nambile Cumber

**Affiliations:** 1 Section for Epidemiology and Social Medicine, Department of Public Health, Institute of Medicine-Master in Global Health, The Sahlgrenska Academy at University of Gothenburg, Box 414, SE-405 Gothenburg, Sweden; 2 Postdoctoral Fellow, Centre for Health Systems Research & Development, University of the Free State, Bloemfontein, South Africa; 3 Office of the Dean, Faculty of Health Sciences, University of the Free State, Bloemfontein, South Africa; 4 School of Health Systems and Public Health, Faculty of Health Sciences, University of Pretoria, Pretoria, South Africa

**Keywords:** COVID-19, response, street children

## To the Editors of The Pan African Medical Journal

The unprecedented novel Coronavirus disease, COVID-19, which emerged in December 2019 has been labeled “disease X” by the World Health Organization (WHO) based on its rate and extent of spread as well as harsh ramifications. In response, different nations have adopted different management approaches hinging on various considerations, among them the WHO recommendations. Uganda has incorporated lockdowns, a measure that has affected schools, hospitals, businesses, places of worship, markets, public transport, and a rise in abuse of human rights in the form of police brutality, and domestic violence [1]. However, contextual factors such as socioeconomic realities and poverty pose a different set of problems for the mitigation and suppression of efforts for COVID-19, especially for vulnerable populations. Eventually, the effects of these interventions pitch down to individual citizens but invariably disproportionately affect vulnerable populations such as street children. This commentary examines the effects of mitigation efforts of COVID-19 on street children who roam the streets of Ugandan cities and whose plight barely gets on the research as well as policy agenda [2]. This paper also draws parallels with similar stories from street children in a few other African countries.

According to the Consortium for Street Children (CSC), street children are defined as those who depend on the street with a substantial link to public places for livelihood and work. This population of children and youths is quite dynamic and exact numbers are difficult to establish [3]. It is, therefore, challenging for governments to plan for them. Each child bears a unique story as to why they decide to depend on the street. However, some common reasons include mistreatment at home-34.6%, poverty-25.7%, and the death of parents and guardians-20.6% [2]. Additionally, UNICEF highlights the widespread urbanization which comes with an inflated cost of living outside the realm of affordability for poor families, forcing children to the streets [4]. Street children were first recorded in Uganda in the early 1970s under the existence of a civil war that produced over 800,000 orphans [2]. Despite the fact that Uganda has grown economically and politically, it does not meet the street children´s welfare owing to a lack of government support in the form of budgetary allocations. In comparison, only 0.5% of the street children in Cameroon report having received some form of government support [5]. They also receive aid from well-wishers and NGO´s for example the recent United Nations distribution of 1200 aid kits for street children in Senegal, a country with more than 30000 street children [6,7]. However, such aid is not sustainable and does not adequately meet their needs. These facts reflect how this demographic is often neglected. Below, we discuss some of the responses where street children in Uganda are delineated during the responses to COVID-19.

First, whereas the rest of the population in homes access information presented in technological devices such as radios, televisions, phones, and the internet, street children do not have these. Their primary source of information is from street workers who are currently not working [8,9]. Some groups have advocated for printing information that these children could read but this is not on the priority agenda. Street children, therefore, remain uninformed and can be a drawback to the efforts against the continuous spread of the virus. Likewise, documented literacy levels among street children show that 25.5% of them do not have formal education and over 70% have at most elementary school education [4]. This observed low literacy levels is another impediment to the interpretation of the little health information they can access. The children are thus left misinformed and relying on rumors and myths and this has been highlighted in other African countries like Kenya and Ghana [9].

Second, due to the characteristics of their lifestyle, street children often reside in meager accommodation in groups, with no access to clean water, food, or sanitation facilities. These children do not like being on the streets as underscored by a recent interview with a street child in Mombasa, Kenya who revealed that he would rather be arrested during this crisis because there is guaranteed food and shelter in prison [7]. But some street children will avoid incarceration during lockdown enforcement and as they avoid being jailed, they also either spread the disease or increase their own risk of morbidity. Additionally, while it is clear that the WHO mitigation strategies such as staying at home and personal distancing and hygiene are crucial for COVID-19 prevention, this is impractical for street children given the harsh realities they face. Another way in which lockdowns due to COVID-19 has affected street children is to deprive them of their income. Their main economic activity is begging and with lockdowns in place, they risk dying of hunger rather than Coronavirus since the people from whom they beg are all under lockdown and off the streets [7].

Lastly, 50% of street children are exposed to violence including sexual violence on the streets [8]. These rough experiences tend to make them insubordinate and hence likely to remain defiant even when advised about possible ways to avoid the virus. According to Cumber [5], 44.9% of these children have lived on the street for only 7-12 months and 88.2% had no contact with their families. However, the current unpalatable conditions on the street during COVID-19 lockdown with nothing to eat may force them to go back to their abusive families from which they escaped. This may expose them to repeated bouts of violence. On the contrary, more children may get on the streets after lockdowns as poverty and orphanage levels increase following the intentional recessions in the economy and deaths of parents from the virus [7]. There have been positive stories of this pandemic having reunited some street children with their families in the neighboring Kenya courtesy of the philanthropic work of a nun [10]. As encouraging as this sound, no amount of philanthropy could save all the numerous street children in Uganda and the rest of the African continent. The responsibility rests with the governments.

## Conclusion

Measures against COVID-19 have made drastic changes to global public health. However, they have punctuated the problems of low- and middle- income Sub-Saharan African Countries, Uganda inclusive, with already existing vulnerable populations. The limited literature on street children in Uganda emphasizes a need for their important inclusion in policy and research decisions to preserve their lives and wellbeing. This commentary has found paucity in information and research about street children and further research on this topic would bring more conclusions to the true impact of COVID-19 on street children, and thus better considerations.

## Competing interests

The author declares no competing interests.

## References

[1] Institute for African Women In Law (IAWL) (2020). Gender, Police Brutality, and Public Health in Uganda During the COVID-19 Pandemic.

[2] Young L (2004). Journeys to the street: the complex migration geographies of Ugandan street children. Geoforum.

[3] Consortium for Street Children Street children are one of the most vulnerable children on the planet.

[4] UNICEF Evaluation database. 2001 ZIM: A Study on Street Children in Zimbabwe.

[5] Cumber SN, Tsoka-Gwegweni JM (2016). Characteristics of street children in Cameroon: A cross-sectional study. Afr J Prim Health Care Fam Med.

[6] Dogru Alaattin UN distributes aid kits for street children in Senegal. 15.05.2020.

[7] Griffin J (2020). ‘Will we die of hunger?’: how Covid-19 lockdowns imperil street children. The Guardian.

[8] UNICEF Evaluation database: 2001 ZAM: Rapid Assessment of Street Children in Lusaka.

[9] StreetInvest (2020). COVID-19 on the Streets.

[10] Aineah A Kenyan Nun Braving Harsh COVID-19 Times to Sustain Street Feeding Program.

